# Success and failure of endodontic treatment: predictability, complications, challenges and maintenance

**DOI:** 10.1038/s41415-025-8453-5

**Published:** 2025-04-11

**Authors:** Dipti Mehta, Alexandra Coleman, Maria Lessani

**Affiliations:** 41415398817001 Specialist in Endodontics/Consultant in Endodontics, Chequers Endodontic Practice, 7 Chequers Drive, Prestwood, Great Missenden, Bucks, HP16 9DU, UK; King´s College Hospital Dental Institute, Department of Restorative Dentistry, Bessemer Road, London, SE5 9RW, UK; 41415398817002 Senior Clinical Teacher/Honorary Consultant in Restorative Dentistry, Academic Unit of Restorative Dentistry, School of Clinical Dentistry, The University of Sheffield, Claremont Crescent, Sheffield, S10 2TA, UK; 41415398817003 Specialist in Endodontics/Part-Time Clinical Lecturer, Eastman Dental Institute, UCL, London, UK; Part-Time Private Practice, Endoclinic, North London, UK

## Abstract

The fundamentals of successful endodontic treatment are an awareness of the aetiology of the disease process and an understanding of factors that affect outcome. This paper aims to outline the prognostic factors found in the endodontic outcome literature to facilitate options appraisal and predictable treatment delivery. We will discuss pre-treatment, treatment and post-treatment factors. In summary, the significance of infection control throughout treatment, provision of an adequate coronal seal and appropriate restoration of the root-filled tooth are highlighted.

## Key points

- Knowledge of the aetiology of endodontic disease and the factors affecting the outcome of root canal treatment are the key to decision-making and the provision of predictable treatment.
- Factors related to the extent of microbial contamination of the root canal space and our management of this have a significant impact on success.
- Provision of an adequate coronal seal and the appropriate restoration of the tooth are key contributors to long-term success.

## Introduction

An understanding of the factors that will affect the outcome of endodontic treatment is essential in the decision-making process when planning endodontic treatment. This paper aims to inform clinicians of the fundamentals of successful endodontic treatment in a manner that will enhance predictable delivery.

## How do we define endodontic success?

**Fig. 1:**
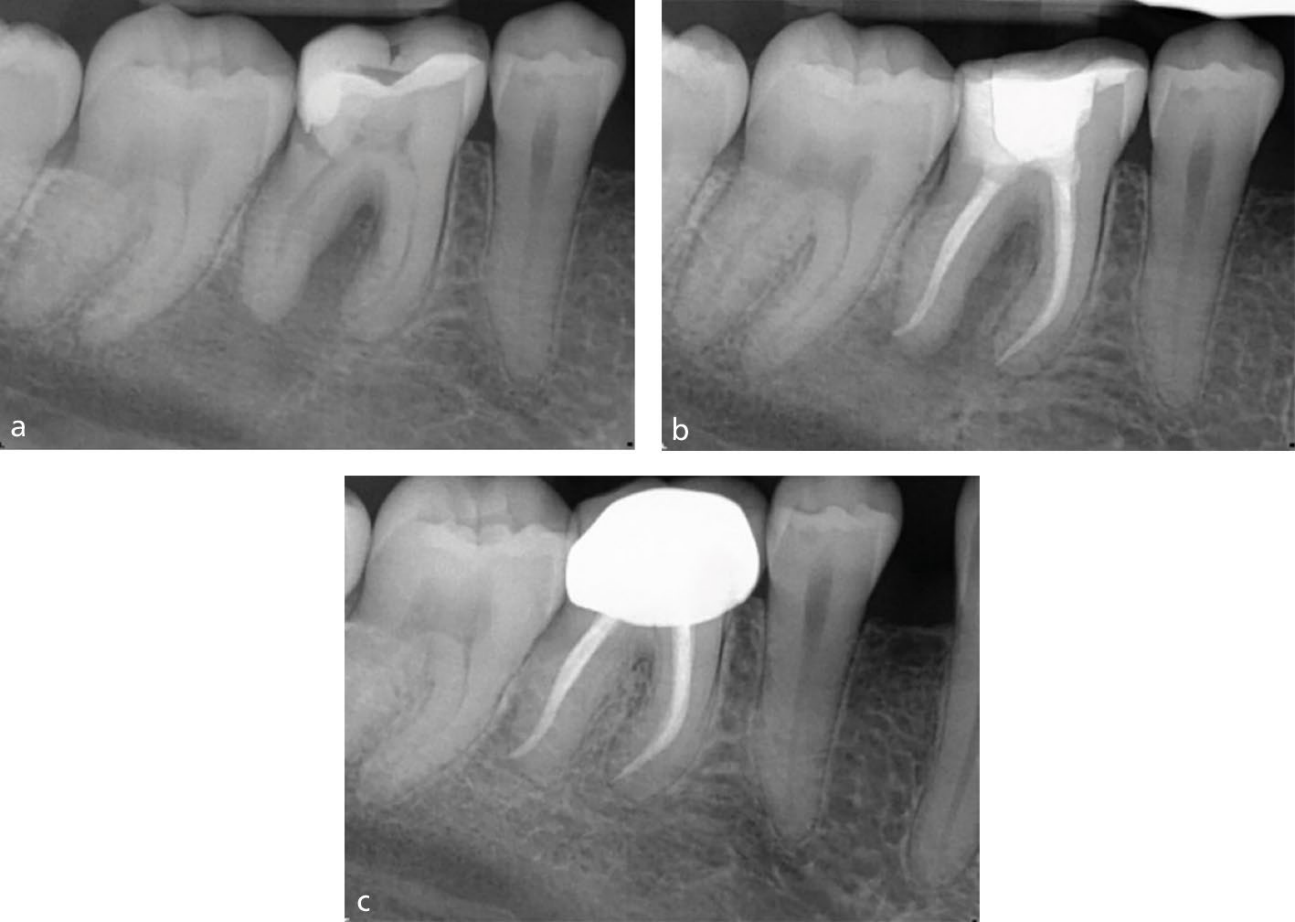
Complete periapical healing. a) Pre-operative radiograph. b) Post-obturation radiograph. c) Two-year review

Success - also referred to as a favourable endodontic outcome^1^ - is defined as the absence of symptoms and clinical signs of disease, such as mobility, sinus tract or probing defect, with no loss of function. Radiographically, the apical periodontal ligament space should be intact with resolution of any previous periapical radiolucency, indicating bony healing (Fig. 1).

Several outcome measures are used when evaluating endodontic success in clinical studies: tooth survival, clinician-reported outcome measures (CROMs) - clinical and radiographic - and patient-reported outcome measures (PROMs).^2^ These are explained in Table 1.

**Table 1:** Outcome measures of success from the literature

**Outcome measure**	**Description**
Tooth survival	The retention of the tooth regardless of disease status with no further intervention, such as root canal retreatment, root-end surgery or extraction
CROMs: clinical success	Functional tooth in the absence of clinical signs of persistent disease including pain, swelling, sinus tract, with normal periodontal probing depths and normal mobility
CROMs: radiographic success	Complete resolution of preoperative periapical radiolucency at recall (strict criteria) Either complete resolution or reduction of the preoperative periapical radiolucency at recall (loose criteria)
PROMs	No pain Tooth saved Functional

Most of the literature provides us with data regarding the factors which affect endodontic success (CROMs) and survival. Traditionally, the focus has been on clinical and radiographic success,^3^ inferring a positive biological response to treatment. In recent times, emphasis has been given to tooth survival.^4^ Use of the latter as an outcome measure gained popularity when comparisons to implants were made.^5^

The perception of success can vary among patients and clinicians. PROMs are linked to an understanding of patient and society perspectives, thus the impact of treatment on a patient's quality of life has been identified as a key outcome measure. The lack of good-quality evidence in this important area of understanding the patient's perception of endodontic outcome is related to the poor validity of the measurement tools, making conclusions difficult to draw.^6^^,^^7^

## Variations in the existing literature

One of the main criticisms of the existing endodontic outcome studies is their heterogeneity, making strong conclusions more challenging to draw.^2^ Some of the variations in the existing endodontic outcome literature are highlighted in Table 2.

**Table 2:** Variations in outcome studies

	**Variations encountered**
Study design	Case series Cohort studies: retrospective and prospective Randomised clinical trials
Operator	Undergraduate or postgraduate students General dental practitioners Specialist in endodontics Mix of clinicians
Clinical Protocol	Use of dental dam Variations in instrumentation techniques (stainless steel versus nickel-titanium), irrigants, obturation materials and coronal seal placement Single visit versus multiple visits
Outcome measures	Clinical and radiographic success Radiographic only Survival Functional survival Use of periapical radiographs or CBCT scans
Follow-up periods	6 months to 10+ years

As clinicians, we rely on the evidence from the literature to help us in the decision-making process, yet from Table 2, it is evident how variation among studies can make the literature difficult to interpret and apply to our clinical setting. The need to understand the evidence and relate it simply to our patients for decision-making^8^^,^^9^^,^^10^ is imperative for informed consent.

## Endodontic success rates in the literature

The endodontic outcome literature is composed of mainly cohort studies, with few randomised clinical trials. Systematic reviews with meta-analysis of these studies are accepted as the best level of evidence available to us. Table 3 summarises the most quoted success and survival rates for root canal treatment and root canal retreatment outcomes from the Eastman group.^11^^,^^12^^,^^13^

**Table 3:** Success rates for non-surgical endodontic treatment and retreatment

**Procedure**	**Outcome measure**	**Evidence**	**Rate**
Root canal treatment	Clinical and radiographic success	Ng *et al.* 2007	75% strict criteria 85% loose criteria
Root canal retreatment	Clinical and radiographic success	Ng *et al.* 2008	77% strict and loose criteria
Root canal treatment	Tooth survival	Ng *et al.* 2010	86% 2-3 years 93% 4-5 years 87% 8-10 years

Endodontic treatment failure is most often related to intra-canal infection via a persistent microbial biofilm^14^ or recontamination of the root canal system through coronal leakage or crack development.^15^^,^^16^ When discussing the individual prognostic indicators which influence success of endodontic treatment, we can broadly divide these into pre-treatment, treatment and post-treatment factors.

## Pre-treatment factors

### Patient factors

Patient-related factors, such as age and sex, have not been shown to have a significant effect on treatment outcome.^17^ The effect of medical history, particularly in relation to conditions affecting inflammatory response, has been studied, with systematic reviews indicating a negative effect of diabetes on periapical healing outcome;^18^^,^^19^ however, the limited number of studies included in the reviews means the results must be interpreted with caution.

### Tooth factors

#### The periapical lesion

**Fig. 2:**
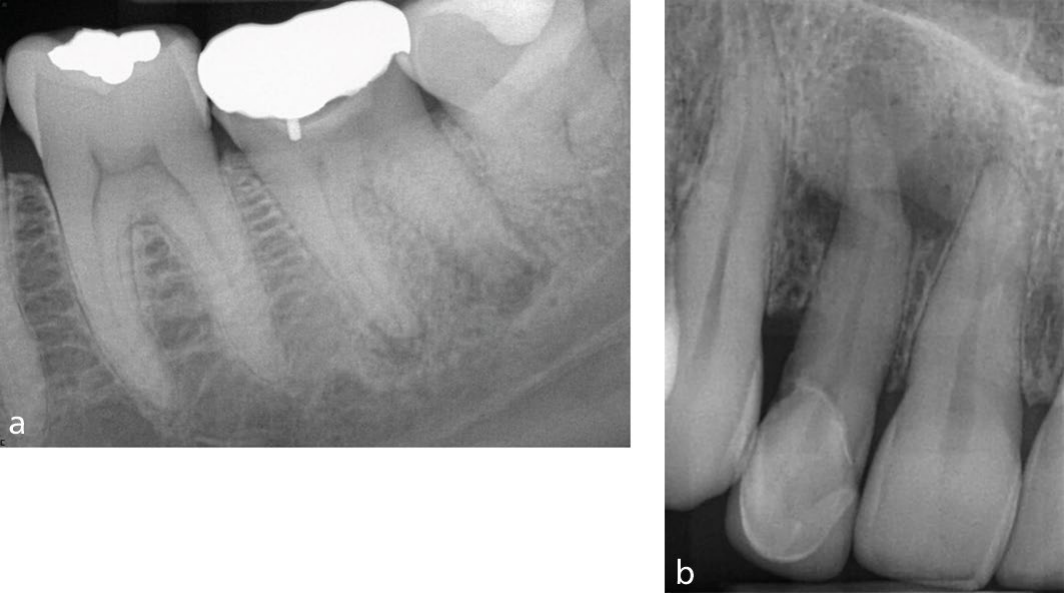
Periapical radiographs with differing PA lesion size. a) Lower left first molar with periapical radiolucencies. b) Upper right lateral incisor with a large periapical radiolucency

The absence of a periapical (PA) lesion is a positive prognostic factor.^12^^,^^20^^,^^21^ In contrast, the presence of a lesion has a significant negative effect on healing outcome.^12^^,^^20^ This can be explained by an understanding of the development of apical periodontitis. A PA lesion forms in the presence of bacterial contamination of the root canal space.^22^ The presence of an intra-radicular biofilm within the anatomical complexities is challenging to remove,^14^ resulting in a negative effect on PA healing. The larger the lesion (Fig. 2), the more complex the infection,^23^ which is reflected in a less favourable outcome in teeth with large PA areas.^20^ In one prospective study, Ng *et al*. (2011) concluded that ‘the odds of success of treatment were found to decrease by 14% for every 1 mm increase in diameter of the preoperative lesion'.^20^ In Figure 2, the upper right lateral incisor will have a reduced prognosis compared with the lower left first molar, which has a smaller periapical lesion.

#### Presence of preoperative sinus

**Fig. 3:**
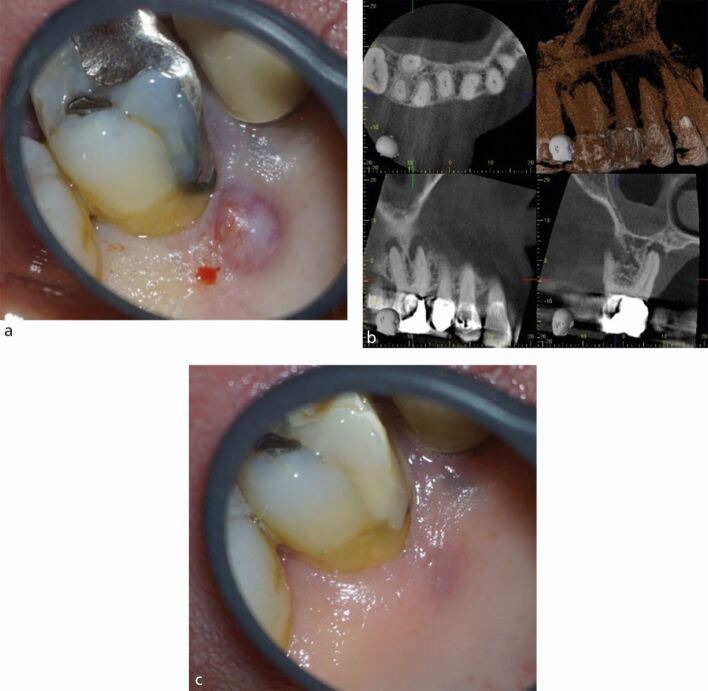
Clinical and radiographic appearance of a sinus tract. a) Upper right first molar with a preoperative palatal sinus tract. b) CBCT scan showing perforation of the palatal cortical plate. c) Postoperative sinus healing

In the context of endodontics, a sinus tract develops following periapical inflammation which has resulted in loss of at least some of the adjacent cortical plate or if the root is outside the bony envelope (Fig. 3). The presence of a sinus has been linked to both a reduced periapical healing outcome^20^ and a poorer survival rate.^16^ A sinus has been linked to a possible entry point for extra-radicular infection.^24^

#### Presence of a crack

A discussion on the aetiology, diagnosis and management of cracks is beyond the scope of this paper; however, their presence or inferred presence is considered a negative prognostic factor for survival.^16^^,^^25^

**Fig. 4:**
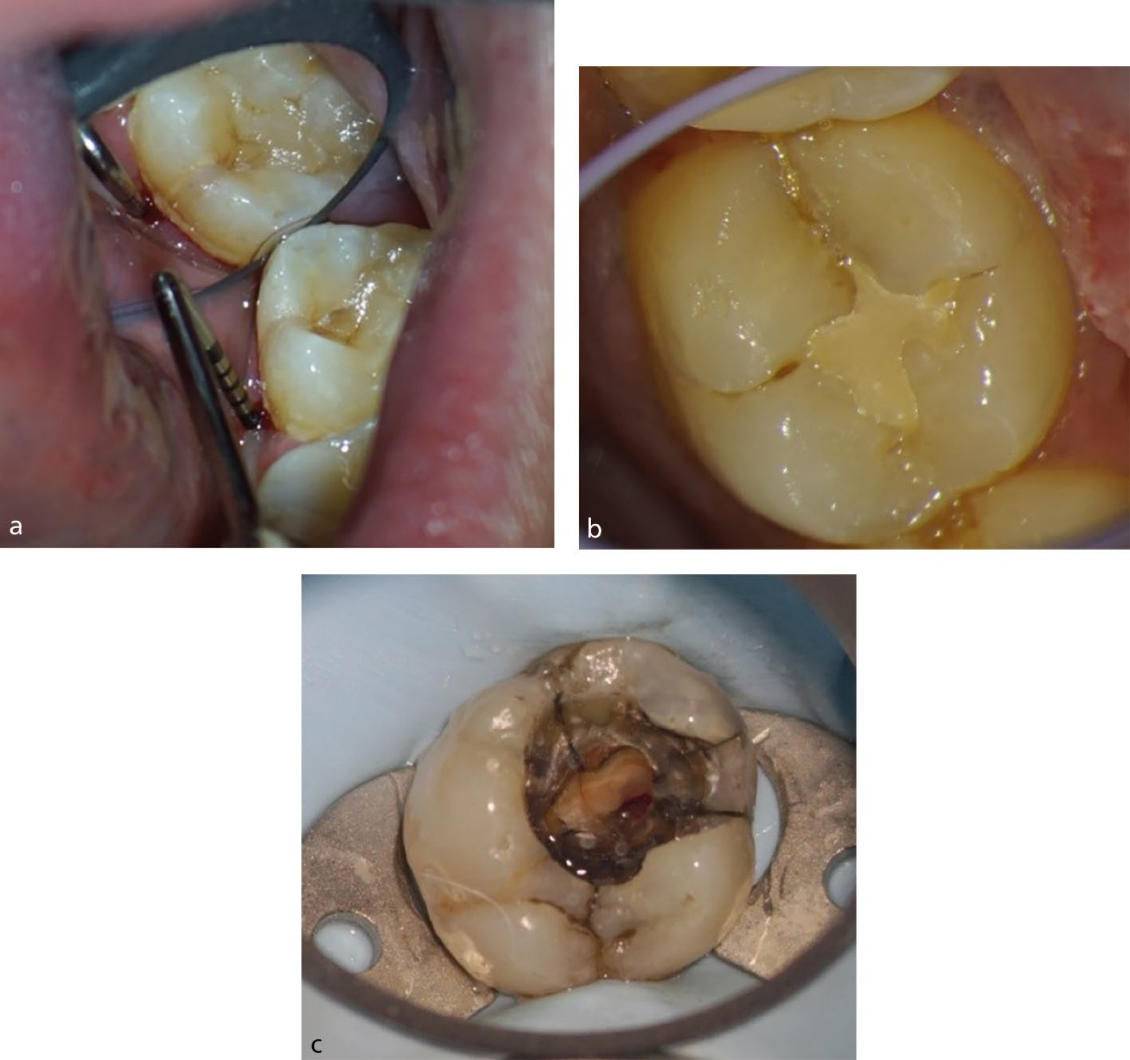
Presentations of cracked teeth. a) Narrow deep pocket adjacent to mid-lingual crack on the lower left second molar tooth. b) Debris housing crack affecting the distal marginal ridge of the lower right second molar tooth. c) Crack along the mesial wall of an upper left first molar

The presence of a crack can be a route of microbial ingress, as well as affecting the structural integrity of the tooth. A sign of crack propagation - often considered pathognomonic of cracks affecting the root - is the presence of a localised narrow pocket (Fig. 4). The pocket results from propagation of a crack onto the root surface causing an endodontic-periodontal lesion (EPL) with root damage.^26^ The presence of the pocket is considered a negative prognostic indicator for tooth survival, along with the terminal position of the tooth and extension of the crack into the canal orifices.^16^^,^^25^

#### Tooth restorability

The restorative status of a tooth requiring endodontic treatment will influence the ability to achieve an optimal coronal seal both during and after treatment. The importance of a good-quality coronal restoration has been highlighted in terms of PA healing^12^^,^^20^^,^^21^ and tooth survival.^16^ Restorability will also influence the ability to achieve dental dam placement and adequate isolation during endodontic treatment. Most teeth requiring endodontic treatment will have a history of caries, large restorations, or cracks/fractures, compromising the amount of remaining tooth structure.^27^ Restorability assessment before proceeding with treatment is therefore an integral part of endodontic care. Removal of the existing restoration allows assessment of the feasibility of an adequate coronal seal and facilitates planning of the final definitive restoration that will address occlusal form, function and aesthetics.

Indices can be used to aid assessment of restorability.^28^^,^^29^ The Dental Practicality Index^29^ was used in a recent study looking at tooth survival in root canal-retreated posterior teeth. Following root canal retreatment of posterior teeth, Al-Nuaimi *et al*. (2020)^30^ identified that when less than 29.5% of tooth structure was remaining, the percentage of extractions was three times higher compared to teeth with more than 29.5% tooth structure remaining.

Historically, the importance of the marginal ridges for support and strength has been identified; therefore, loss of marginal ridges is considered to contribute to a weaker structure. Tooth position is another consideration, as terminal or lone-standing teeth show poorer survival, being at a greater risk of fracture due to increased occlusal forces.^17^

An optimal ferrule is important for the success of an indirect restoration following endodontic treatment. A ferrule is defined as an encircling band of cast metal (or restorative material) around the coronal surface of the tooth. The absence of an adequate ferrule effect reduces survival of both the restoration and the root-filled tooth.^31^ In a literature review on the ferrule effect, Juloski *et al*. (2012)^32^ concluded that the presence of a 1.5-2 mm ferrule has a positive effect on fracture resistance of root-filled teeth and that an incomplete ferrule is considered better than a complete lack of ferrule. Therefore, when planning the post-endodontic restoration, an assessment of height and thickness of remaining supragingival tooth structure at each tooth surface should be made to inform the possible presence or absence of ferrule and whether this ferrule will be complete or incomplete. A lack of sufficient ferrule should therefore make you question whether the tooth is indeed restorable and whether an optimum coronal seal can be achieved without encroaching upon the supracrestal tissue attachment.

#### Periodontal status

EPLs occur because of a pathological communication between the pulpal and periodontal tissues at a given tooth that may occur in acute or chronic form.^33^ The most recent update in classification of EPLs highlighted that these should be classified according to signs and symptoms that have a direct impact on their prognosis and treatment. As such, EPLs are classified as: 1) EPL with root damage; 2) EPL in a periodontitis patient, with no root damage; and 3) EPL in a non-periodontitis patient, with no root damage.^33^

EPLs with root damage, including cracks and perforations, are discussed elsewhere in this paper. In the absence of root damage, the prognoses of EPLs are considered more variable. Periodontal status impacts prognosis due to changes in the oral microbiome of patients with unstable periodontitis.^26^ A detailed periodontal examination is a prerequisite for an accurate diagnosis and treatment plan for an EPL.^26^ In a periodontally unstable patient, the prognosis is worse than in a periodontally stable or non-periodontitis patient.^26^ However, there is long-term evidence demonstrating statistically comparative outcomes of periodontal regenerative surgery (± endodontic treatment) versus extraction and tooth replacement in teeth with attachment loss to the apex in Stage III or IV periodontitis.^34^ This randomised clinical trial also showed the total mean cost of treatment over the observation period was significantly lower for teeth retained with regeneration, thus, supporting the retention of teeth with severe periodontal attachment loss to the apex.

### Treatment factors

When considering treatment factors that impact success, we are broadly considering the following: infection control during treatment; tooth anatomy; factors related to chemo-mechanical preparation and obturation of the canal space; and the avoidance of iatrogenic errors.

#### Infection control during root canal treatment

The use of dental dam during endodontic treatment is mandatory from a patient safety, as well as infection control standpoint.^35^ Its impact on achieving good endodontic outcomes has been shown.^36^^,^^37^ The European Society of Endodontology's (ESE) S3-level clinical practice guidelines recommend ‘a meticulous aseptic technique and optimal surgical field including the use of dental dam'.^38^ A study^39^ assessing the clinical outcome of endodontically treated teeth in a specialist practice found 17.6% of teeth without a preoperative PA lesion as confirmed by cone beam computed tomography (CBCT) scan developed a lesion at 12-month review. The implication was microorganisms contributing to the lesion may have been introduced during endodontic treatment.^39^

Clinical outcome studies testing this implication have found an enhanced infection control protocol improves PA healing,^40^^,^^41^ highlighting the need for careful infection control while performing treatment. Although the protocol was much stricter in the Zahran *et al*. 2021 study,^40^ Table 4 summarises a set of recommended practical steps to limit canal contamination during treatment using the protocols from both studies,^40^^,^^41^ highlighting aseptic handling of instruments and material by all the dental team.

**Table 4:** Enhanced infection control protocol recommendations based on outcome studies^40^^,^^41^

**Recommended measures to reduce contamination during endodontic treatment**	
Following examination and local anaesthesia	Don a new pair of gloves to avoid contamination from the oral cavity
Dental dam placement	Disinfect dam by wiping with a sterile gauze or cotton pellet soaked in 2.5% sodium hypochlorite or alcohol
Chemo-mechanical preparation	Use of sterile instruments for endodontic treatment
	Work through a reservoir of sodium hypochlorite in the pulp chamber throughout treatment
	Use of sterile endodontic files
	Clean contaminated file flutes filled with sterile gauze or sponge soaked in sodium hypochlorite/alcohol, to avoid microbial transfer between canals as well as maintaining the cutting efficiency of the file
Intra-operative radiographs	Change/decontaminate gloves after taking radiographs
Obturation	Change/decontaminate your gloves before obturation
	Sterile paper points to dry the canals
	Disinfect the gutta percha points by soaking in sodium hypochlorite for 15 minutes. Wipe with a sterile gauze
	If injecting the sealer directly into the canal, wipe the tip of the sealer with a sterile gauze soaked in sodium hypochlorite
Restoration	Place a well-sealing definitive restoration straightaway, if possible, to avoid recontamination

#### Tooth anatomy

**Fig. 5:**
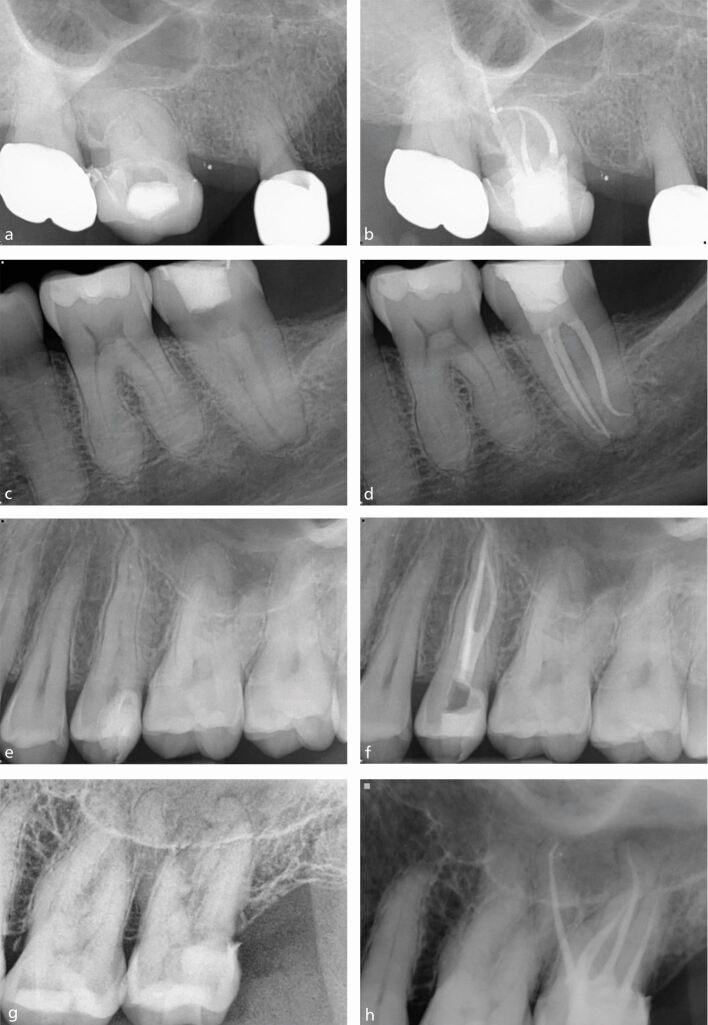
Pre- and post-operative radiographs of teeth with complex anatomy. a, b) Upper right first molar with severe curvature. c, d) Lower left second molar with acute distal curve. e, f) Three-rooted upper left second premolar. g, h) Four-rooted upper left second molar with five canals

Although systematic reviews tell us tooth type does not influence the odds of success,^21^ one of the widely accepted causes of endodontic treatment failure is untreated anatomy housing persistent endodontic infection. Common sense dictates a sound knowledge of endodontic anatomy and identification of teeth/roots with multiple canals should facilitate treatment success. Some of the anatomical variants to consider include additional canals (classically the presence of a second canal in the mesio-buccal root of an upper molar or a second lingual canal in lower incisors), additional roots (three-rooted premolars, radix entomolaris/paramolaris), anatomical complexities (C-shaped canals, dens invaginatus, isthmus between canals, apical delta, lateral canals) and extremes of canal curvature (Fig. 5). Knowledge of the anatomical variations and how to identify them is an essential part of managing the endodontic infection. We can use various methods of identifying additional roots and canals via clinical and radiographic assessment.

#### Chemo-mechanical preparation

Studies demonstrate 43-49% of the canal walls remain untouched during mechanical instrumentation of the root canal;^42^ therefore, additional chemical disinfection of the anatomy is essential to manage the microbial infection. The maintenance of canal patency is one of the main canal preparation factors affecting success.^16^^,^^20^ Having patency is synonymous with mechanical access to the full length of the canal, as it is defined as the passage of a small file through the apical foramen during canal preparation. Apical extent of the preparation is the other prognostic factor considered to be important for successful treatment.^20^ Both factors address the aim of mechanical preparation, which is to facilitate disinfection by allowing irrigant access to the apical infection.

Other factors that have been studied as having an impact on outcome are preparation size and taper. Neither has been shown to influence healing.^12^^,^^20^^,^^21^ In the current era of smaller preparations, it may be argued optimal fluid dynamics cannot be achieved if the size and taper of the preparation does not facilitate the delivery of the irrigant needle to within 1 mm of the preparation length^43^^,^^44^ due to the vapour lock effect.

When considering irrigation, sodium hypochlorite is universally supported as the irrigant of choice during non-surgical root canal treatment.^35^^,^^44^ There is some evidence against the use of chlorhexidine for irrigation.^20^ This may be related to the production of a precipitate (para-chloroalanine) when sodium hypochlorite and chlorhexidine are combined. The by-product is carcinogenic and cytotoxic; therefore, combination of the two irrigants is not advised. In addition, if used as a sole irrigant, chlorhexidine lacks the tissue dissolution effect of sodium hypochlorite. Irrigation with ethylenediaminetetraacetic acid (EDTA) is recommended to remove the smear layer created during canal preparation.^44^ The improvement in treatment outcomes is particularly highlighted for retreatments when EDTA is used as a penultimate rinse.^20^ EDTA facilitates breakdown of the microbial biofilm,^45^ as well as allowing access for the sodium hypochlorite to the tubular infection following smear layer removal, resulting in improved outcomes.

Static needle irrigation is the most commonly used irrigant delivery mechanism However, laboratory studies have highlighted its limitations.^44^ To overcome these limitations, activation of the irrigant solutions via manual dynamic agitation with a gutta-percha cone or the use of sonic devices, ultrasonic devices and lasers, has become popular. Although the limitations of the clinical outcome studies assessing effectiveness don't provide us with strong evidence for their clinical efficacy, lab-based studies continue to support biofilm disruption with their use and so these methods are commonly used in endodontics.^44^

#### Canal obturation

**Fig. 6:**
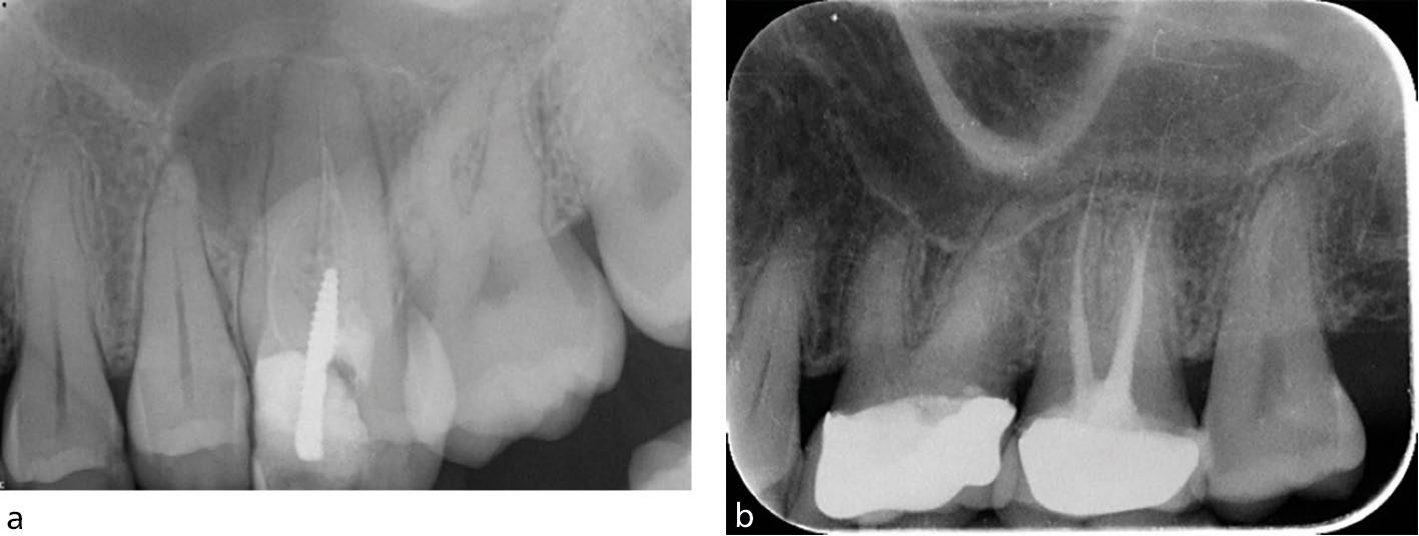
Radiographs showing obturation errors. a) Upper left first molar with a poorly compacted short root filling. b) Upper left first molar with an overextended root filling

The quality of the root filling is judged radiographically by the compaction of the material and its length in relation to the root apex. The absence of voids, the extension of the material to within 2 mm of the radiographic apex and the absence of root filling extrusion (Fig. 6) are all significantly related to a positive treatment outcome.^20^^,^^21^ To dissect the reasons behind this, both the presence of voids and inadequate extension of the root filling are likely related to the residual biofilm not being sufficiently entombed, hence allowing persistence of the intra-radicular infection.^14^ The overextension of the material beyond the apex may result in periapical inflammation, firstly due to a potential foreign body reaction, and secondly from microbial contamination of the gutta-percha cone.^24^ Overextension often occurs when the canal has not been appropriately shaped and the size of the apical foramen inadequately gauged. This type of ‘overfill' may be considered a surrogate measure of how diligently the treatment may have been performed.

#### Role of radiographs during treatment

Periapical radiographs are used for diagnosis of endodontic disease, quality assurance during treatment and as a baseline record post-treatment to monitor healing outcome. The importance of preparation and obturation length have been highlighted above. Intra-operative radiographs allow verification of these parameters to facilitate the delivery of predictable endodontic treatment. Table 5 lists the possible radiographic images that may be taken, their role in treatment, and highlights their need based on existing guidelines. The intra-operative images should be taken with the dental dam in situ with the use of appropriate endodontic film holders.

**Table 5:** Role of radiographs during endodontic treatment

**Image taken**	**Role of image**	**Do I need to take it?**
Preoperative	Pre-treatment control image to facilitate diagnosis and treatment planning	As a record of the tooth before treatment, this film is the pretreatment control** ESE 2006 Duncan *et al.* 2023
Working length	Ideally taken with a file at the apex locator zero reading or in the absence of a zero reading to the apical limit of the file. The image allows verification of the length in relation to the radiographic apex before preparation	Useful radiograph, especially when you are not confident of your apex locator reading* ESE 2006
Master apical file	Taken with a file at the preparation length. The image allows verification of the preparation length in relation to the working length radiograph and the subsequent master cone image	Useful as a check on the maintenance of the canal shape during preparation as well as to verify the gauge of the canal^†^ Often skipped for the master cone film^†^
Master cone/cone fit	Taken with the gutta percha cone in place at the preparation length, this image verifies the length of the obturation in relation to the preparation length	Allows visualisation of the apical extent of the root filling before completion, thus allowing for the correction of any length errors* ESE 2006
Mid-fill	Taken mid-way through obturation to check length and compaction of the apical root filling	Useful in open apices/ canals when filling with hydraulic calcium silicate-based cements to assess length of placement and presence of voids, thus allowing correction of any errors* Can be used in the same way for gutta percha root fillings in wide canals to check adequate compaction of the root filling before restoration^†^
Postoperative	Taken following canal obturation and ideally with the direct coronal restoration in place	As a record of the treatment, this film acts as a radiographic post-treatment baseline** ESE 2006
Key ** = Required * = Highly recommended At least one length check radiograph prior to completing the obturation is strongly recommended ^†^ = Optional		

#### Avoidance of iatrogenic errors (perforation, separated instrument)

Iatrogenic errors can negatively affect the outcome of treatment for two main reasons. Firstly, they may prevent or limit the disinfection of the canal anatomy fully and secondly, they may affect the structural integrity of the tooth. The main errors during treatment are perforation, ledge formation or blockage and instrument separation.

##### Perforation

The presence of a perforation significantly affects the success of treatment, particularly when the perforation was at the coronal or mid-root level.^20^^,^^46^ It is likely that bacterial contamination, as well as the weakening effect of dentine loss at this level, contributes to the poorer outcomes. The size and timing of repair are also relevant for the same reasons.^47^

##### Blocked/ledged canals

A short root filling can be considered synonymous with a blocked or ledged canal. The reduced outcomes in such teeth can be attributed to the persistent intra-radicular infection. This is particularly a concern for retreatment cases, as the presence of an intra-operative canal blockage is particularly significant here.^20^

##### Instrument separation

**Fig. 7:**
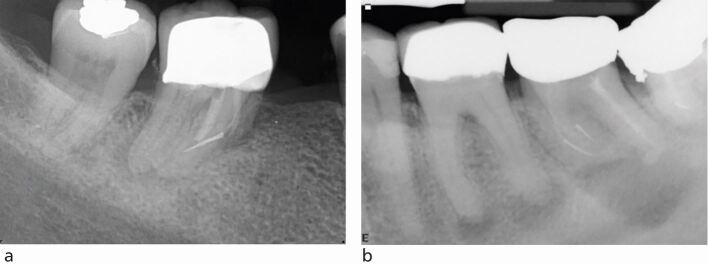
a, b) Radiographs showing fractured instruments in lower molars

Instrument separation can be distressing for both the patient and the clinician performing the treatment. If the instrument can be successfully removed or bypassed, there is no negative effect on treatment outcome.^48^ However, if this is not feasible and a periapical lesion is present, the apical microbial infection becomes difficult to access, and so the outcome is less predictable. The radiographs in Fig. 7 demonstrate fractured instruments *in situ*.

## Post-treatment factors

The restoration of the root-filled tooth is an essential component of root canal treatment.^31^ The key functions of the coronal restoration of a root filled tooth are listed in Box 1.

Upon completion of endodontic treatment, a good-quality coronal restoration is a positive predictor of both periapical healing^12^^,^^20^^,^^21^ and tooth survival.^16^ A systematic review looking at the impact of coronal restoration versus quality of root canal treatment concluded that coronal seal was as important as the quality of the endodontic treatment in terms of treatment success.^49^ The definitive coronal restoration should be provided as soon as possible upon completion of endodontic treatment. There is evidence of an increased failure rate of endodontic treatment with temporary restorations.^50^

A root-filled tooth is at risk of structural failure due to loss of tooth structure, as well as loss of proprioceptive function;^51^ therefore, the decision whether to provide a restoration which provides cuspal coverage requires consideration. Increasing loss of tooth structure results in increasing cuspal deflection and risk of fracture,^52^ and the loss of a marginal ridge has a significant impact on tooth strength.^53^ As previously mentioned, the weakening effect of caries, trauma, cracks, or previous restorations on teeth requiring endodontic treatment, may increase their fracture risk.^27^ Studies have shown that cuspal coverage restorations significantly improved survival of the root-filled tooth.^54^^,^^55^^,^^56^ A recent study identified that root-filled molars with a direct restoration demonstrated a significantly higher frequency of extraction over a period of ten years compared with those restored with an indirect restoration.^57^

When considering the restoration of a root-filled tooth and whether to provide cuspal coverage, both the British Endodontic Society's *Guide to Good Endodontic Practice*^58^ and the ESE's position statement^31^ recommend that each case should be considered individually. Loss of proximal walls is a strong indication of the need for cuspal coverage.^31^^,^^58^ In addition to the amount of tooth structure remaining, other factors to be considered for the need for cuspal coverage are tooth position, adjacent contacts and occlusal forces.^31^ Factors such as loss of proximal contact, terminal tooth in the arch or second molar have been associated with an increased risk of failure of root-filled teeth^13^^,^^16^^,^^54^ and therefore would benefit from cuspal coverage.

Restorations should be designed to conserve as much sound tooth tissue as possible^31^^,^^58^ and if cuspal coverage is required, onlay restorations used where appropriate.^31^ When restoring a root-filled tooth with an indirect cuspal coverage restoration, there are several options regarding material of choice, which demonstrate a relatively high level of survival. Within a systematic review, Sailer *et al*. (2015)^59^ identified the following single-crown survival rates at five years: metal ceramic = 94.7%; leucite lithium disilicate reinforced glass ceramic = 96.6%; and densely sintered zirconia = 92.1%. In a prospective study, Passia et al. (2013)^60^ reported a similar five-year survival rate for gold crowns of 92.3%. There is limited evidence regarding the effect of timing when providing cuspal coverage upon completion of endodontic treatment. Within a retrospective study, Pratt *et al*. (2016)^56^ identified that posterior root-filled teeth that received a crown four months after endodontic treatment were extracted at three times the rate of those that received a crown within four months of endodontic treatment. If a decision is made that cuspal coverage is justified, this should be provided as soon as possible after completion of endodontic treatment, provided there are no signs and symptoms from the tooth.

Box 1 Key functions of the coronal restoration of a root-filled tooth

- Provide a coronal seal and prevent reinfection of the root canal space
- Restore form, occlusal stability and interproximal contact points
- Restore function
- Protect residual tooth structure
- Ensure health of periodontal tissues
- Aesthetics

## Conclusion

The value of maintaining natural teeth for functional and aesthetic reasons through endodontic treatment has become well-understood by patients. Despite the limitations of the existing outcome studies, the evidence supports the retention of teeth via endodontic treatment. Predictability of root canal treatment involves identification of the prognostic factors and understanding their perceived impact on the outcome. Fundamentally, factors related to infection control throughout treatment, a good coronal seal and provision of an optimal definitive restoration are key contributors to successful root canal treatment.
